# Acute Low-Intensity Aerobic Exercise Modulates Intracortical Inhibitory and Excitatory Circuits in an Exercised and a Non-exercised Muscle in the Primary Motor Cortex

**DOI:** 10.3389/fphys.2019.01361

**Published:** 2019-11-07

**Authors:** Yudai Yamazaki, Daisuke Sato, Koya Yamashiro, Saki Nakano, Hideaki Onishi, Atsuo Maruyama

**Affiliations:** ^1^Major in Health and Welfare, Niigata University of Health and Welfare, Niigata, Japan; ^2^Institute for Human Movement and Medical Sciences, Niigata University of Health and Welfare, Niigata, Japan; ^3^Department of Health and Sports, Niigata University of Health and Welfare, Niigata, Japan; ^4^Field of Health and Sports, Major in Health and Science, Niigata University of Health and Welfare, Niigata, Japan; ^5^Department of Physical Therapy, Niigata University of Health and Welfare, Niigata, Japan; ^6^Graduate School of Medical and Dental Sciences, Kagoshima University, Kagoshima, Japan

**Keywords:** low-intensity aerobic exercise, transcranial magnetic stimulation, intracortical inhibitory circuits, intracortical excitatory circuits, spinal excitability

## Abstract

Recent studies have reported that acute aerobic exercise modulates intracortical excitability in the primary motor cortex (M1). However, whether acute low-intensity aerobic exercise can also modulate M1 intracortical excitability, particularly intracortical excitatory circuits, remains unclear. In addition, no previous studies have investigated the effect of acute aerobic exercise on short-latency afferent inhibition (SAI). The aim of this study was to investigate whether acute low-intensity aerobic exercise modulates intracortical circuits in the M1 hand and leg areas. Intracortical excitability of M1 (Experiments 1, 2) and spinal excitability (Experiment 3) were measured before and after acute low-intensity aerobic exercise. In Experiment 3, skin temperature was also measured throughout the experiment. Transcranial magnetic stimulation was applied over the M1 non-exercised hand and exercised leg areas in Experiments 1, 2, respectively. Participants performed 30 min of low-intensity pedaling exercise or rested while sitting on the ergometer. Short- and long-interval intracortical inhibition (SICI and LICI), and SAI were measured to assess M1 inhibitory circuits. Intracortical facilitation (ICF) and short-interval intracortical facilitation (SICF) were measured to assess M1 excitatory circuits. We found that acute low-intensity aerobic exercise decreased SICI and SAI in the M1 hand and leg areas. After exercise, ICF in the M1 hand area was lower than in the control experiment, but was not significantly different to baseline. The single motor-evoked potential, resting motor threshold, LICI, SICF, and spinal excitability did not change following exercise. In conclusion, acute low-intensity pedaling modulates M1 intracortical circuits of both exercised and non-exercised areas, without affecting corticospinal and spinal excitability.

## Introduction

Regular physical activity or aerobic exercise is well-known to increase brain plasticity (Cotman and Berchtold, 2002; Cotman and Engesser-Cesar, 2002; Kramer and Erickson, 2007; Hillman et al., 2008), which is a process indispensable to learning and memory. Previous animal studies have demonstrated that physical activity upregulates the secretion of growth factors including brain-derived neurotrophic factor (BDNF) (Neeper et al., 1995, 1996), insulin-like growth factor 1 (Trejo et al., 2001; Cetinkaya et al., 2013), vascular endothelial growth factor (Latimer et al., 2011) and nerve growth factor (Ding et al., 2004). The effects of these factors involve angiogenesis (Kleim et al., 2002) and neurogenesis (van Praag et al., 1999; Inoue et al., 2015) in the brain, and these molecular and cellular mechanisms contribute to facilitate brain plasticity. In the human-based research of Cirillo et al. (2009), active individuals exhibited higher paired associative stimulation-induced plasticity compared with sedentary individuals. Moreover, structural and functional plastic changes of the brain have been observed in individuals undertaking long-term motor-skills training (e.g., athletes) (Taubert et al., 2015; Moscatelli et al., 2016; Wang et al., 2016; Monda et al., 2017).

Recent studies have reported that acute aerobic exercise enhances neuroplasticity (McDonnell et al., 2013; Mang et al., 2014; Singh et al., 2014b, 2015) and motor learning (Roig et al., 2012; Mang et al., 2014; Snow et al., 2016). The neurophysiological mechanisms of these positive effects may involve modulation of intracortical circuits in the primary motor cortex (M1) induced by acute aerobic exercise. Many previous studies have shown that acute high-intensity-interval or moderate exercise suppresses short-interval intracortical inhibition (SICI) (Singh et al., 2014a; Stavrinos and Coxon, 2017), though Mooney et al. (2016) did not observe such results. On the other hand, long-interval intracortical inhibition (LICI) has been reported to decrease (Mooney et al., 2016) or remain unchanged (Singh et al., 2014a; Stavrinos and Coxon, 2017) after exercise. Although Neva et al. (2017) reported that acute moderate-intensity exercise increases short-interval intracortical facilitation (SICF), Lulic et al. (2017) did not observe any modulation of SICF. Furthermore, intracortical facilitation (ICF) has been shown to increase (Singh et al., 2014a) or decrease (Lulic et al., 2017) in response to acute moderate exercise. These results have also been observed in the M1 hand area, which is not involved in exercise. Thus, aerobic exercise is suggested to modulate intracortical circuits in non-exercised areas. In contrast, late cortical disinhibition did not change after moderate intensity exercise as shown by Mooney et al. (2016).

These previous studies have several limitations. Firstly, there is little evidence that acute low-intensity aerobic exercise modulates M1 intracortical circuits. Yamaguchi et al. (2012) reported that 7 min of low-intensity pedaling exercise caused suppression of SICI in the M1 leg area. In addition, a decrease in SICI after low-intensity aerobic exercise has also been observed in the non-exercised area of the hand (Smith et al., 2014) without the changes to corticospinal excitability (McDonnell et al., 2013; Smith et al., 2014). However, these previous studies investigated only SICI. Although several studies have reported changes to intracortical excitatory circuits following moderate intensity exercise (Singh et al., 2014a; Mooney et al., 2016; Lulic et al., 2017; Neva et al., 2017), whether low-intensity aerobic exercise modulates other parameters of intracortical circuits, particularly intracortical excitatory circuits, remains unclear.

Secondary, only a few studies have investigated the influence of exercise on intracortical circuits in the M1 leg area. Although SICI decreased in the M1 leg area after pedaling exercise (Yamaguchi et al., 2012) or leg press with fatiguing (Takahashi et al., 2011), the evidence is insufficient. Yamaguchi et al. (2012) did not investigate intracortical parameters other than SICI, and Takahashi et al. (2011) studied non-aerobic exercise. More detailed investigations of aerobic exercise-induced modulation of the M1 exercised-leg area are needed.

Lastly, the effect of aerobic exercise on short-latency afferent inhibition (SAI) has not been investigated. The magnitude of SAI depends on the amount of afferent input. In addition, SAI is influenced by cortical excitability in M1 or the primary somatosensory cortex (S1), indicated by the findings that SAI is enhanced following anodal transcranial direct-current stimulation (tDCS) over M1 (Scelzo et al., 2011) and attenuated following cathodal tDCS over M1 (Sasaki et al., 2016) or S1 (Kojima et al., 2015). SAI reflects the activity of central cholinergic and GABAergic neurons (Di Lazzaro et al., 2000b, 2005a,b). It is therefore important to investigate the effect of acute aerobic exercise on SAI to understand the effects on the central nervous system. Therefore, we decided to explore whether acute low-intensity aerobic exercise modulates SAI. To the best of our knowledge, this is the first study to investigate this effect. Previous studies have demonstrated that acute aerobic exercise decreases the activity of GABAergic neurons (i.e., decreases SICI or LICI); therefore, it is possible that acute aerobic exercise modulates SAI.

The aim of this study was to investigate whether 30 min of low-intensity aerobic exercise causes modulation of intracortical circuits in M1 hand and leg areas. We measured SICI, LICI, and SAI to evaluate changes in inhibitory circuits, and ICF and SICF to evaluate changes in excitatory circuits. Based on the results of Yamaguchi et al. (2012) and Smith et al. (2014)—which showed that low-intensity aerobic exercise modulated GABA_*A*_ergic activity in exercised and non-exercised areas in M1—we hypothesized that acute low-intensity aerobic exercise modulates M1 intracortical circuits, particularly inhibitory circuits related to SICI and SAI.

## Materials and Methods

### Participants

In total, we enrolled 22 participants in the present study. Fifteen (eight females, 21.5 ± 1.6 years) took part in Experiment 1, 14 (seven females, 21.1 ± 1.5 years) took part in Experiments 2, 3, and seven of the enrolled participants took part in all experiments. Participants were right-handed. None had a history of neurological or psychiatric disease, and none were taking any medications at the time of the study. Written informed consent was obtained from all participants after full verbal explanation of the experimental protocol, risk and research goal. This study was conducted in accordance with the Declaration of Helsinki, and was approved by the ethics committee of Niigata University of Health and Welfare.

### Experimental Overview

**FIGURE 1:**
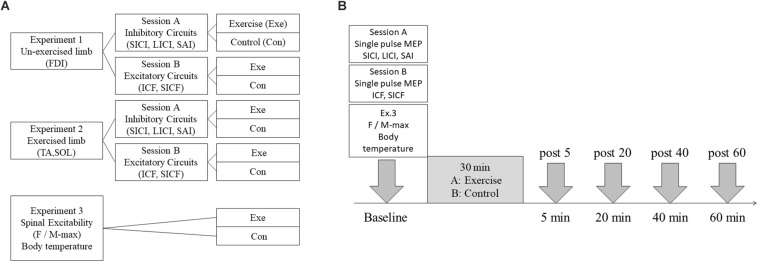
Experimental overview. **(A)** Experimental session. Intracortical circuits in the M1 non-exercised hand and leg areas were measured in Experiments 1, 2, respectively. Spinal excitability in the hand and leg was measured in Experiment 3. In Experiments 1, 2, intracortical inhibitory circuits were assessed by measuring short- and long-interval intracortical inhibition (SICI and LICI), and SAI in session A. Intracortical excitatory circuits were assessed by intracortical facilitation (ICF) and short-interval intracortical facilitation (SICF) in session B. All experiments consisted of exercise and control conditions. Participants performed 30 min of low-intensity aerobic exercise on the recumbent ergometer in exercise conditions, and rested while seated on the recumbent ergometer in control conditions. **(B)** Experimental protocol. In Experiments 1, 2, transcranial magnetic stimulation (TMS) assessments were performed before exercise and 5, 20, 40, and 60 min after 30 min of exercise. In Experiment 3, the F-wave and M-max were measured at the same timing as in Experiments 1, 2.

The overall procedures consisted of two stages: preliminary and main experiments. In the preliminary experiment, participants performed a graded maximal test to determine their individual optimal exercise intensity, defined as 30% of the VO_2__*peak*_. This was based on the classification of physical activity intensity of the American College of Sports Medicine (Thompson et al., 2013). The main experiments were conducted according to a randomized design and consisted of three sessions (Figure 1A). We investigated the change in cortical excitability in M1 non-exercised upper limb and exercised lower limb areas in Experiments 1, 2, respectively. The inhibitory and excitatory circuits of different sessions (inhibitory, session A; excitatory, session B) were measured in both experiments. Spinal excitability in both limbs and skin temperature were measured in Experiment 3. All experiments consisted of exercise and control conditions and were performed at least 3 days apart.

### Graded Maximal Exercise Test

All participants underwent a graded exercise test to exhaustion using a recumbent type ergometer (RT2; Monark, Sweden) to determine VO_2__*peak*_. After a warm-up exercise of 1 min at 60 W, the work rate was increased by 15 W per min in a constant and continuous manner, to exhaustion. The pedaling rate was maintained at 60 rpm. Participant heart rate (HR) and rating-perceived exertion (RPE) were recorded using the Borg scale every 1 min (Borg, 1982). Ventilation parameters, oxygen intake (VO_2_) and carbon dioxide output (VCO_2_) were measured breath-by-breath using a gas analyzer (Aeromonitor AE300; Minato Medical Science, Osaka, Japan) at a sampling rate of 0.1 Hz. The respiratory exchange ratio (R) was calculated as the VCO_2_/VO_2_ ratio, and VO_2__*peak*_ was determined when two of the following criteria were satisfied: R > 1.15, achievement of age-predicted peak HR or RPE of 19 or 20. Values of VO_2__*peak*_ and other respiratory and metabolic parameters at VO_2__*peak*_ are shown in Table 1. To determine the level of exertion required to achieve 30% of VO_2__*peak*_, VO_2_ was plotted against the output power of the ergometer at VO_2__*peak*_ (Wasserman et al., 1973). This showed linear regression of the measured points using the least-square method, and 30% VO_2__*peak*_ was estimated from delta VO_2__*peak*_ (VO_2__*peak*_ - VO_2_ at rest period) for each participant.

**TABLE 1:** Baseline transcranial magnetic stimulation and electric stimulation parameters.

			**RMT (%MSO)**	**AMT (%MSO)**	**Test intensity (%MSO)**	**ES intensity (mV)**
Experiment 1	Session A	EXE	45.7 ± 2.0	36.4 ± 1.5	57.8 ± 2.6	9.88 ± 0.5
		CON	46.0 ± 2.1	36.9 ± 1.3	57.5 ± 2.7	10.49 ± 0.5
	Session B	EXE	47.3 ± 1.8	36.4 ± 1.4	58.6 ± 2.7	
		CON	47.0 ± 2.0	37.2 ± 1.5	58.9 ± 2.6	
Experiment 2	Session A	EXE	46.2 ± 1.4	34.2 ± 1.3	56.1 ± 0.7	18.57 ± 0.9
		CON	46.2 ± 1.3	33.4 ± 1.2	55.4 ± 1.5	18.93 ± 1.0
	Session B	EXE	45.9 ± 1.9	32.7 ± 1.4	55.8 ± 1.6	
		CON	46.3 ± 1.8	32.3 ± 1.4	56.3 ± 1.8	
Experiment 3	Hand	EXE				20.54 ± 0.9
		CON				19.67 ± 0.8
	Leg	EXE				24.14 ± 0.7
		CON				23.52 ± 0.6

*Data are presented as mean ± standard error. RMT, resting motor threshold; MSO, maximum stimulus output; AMT, active motor threshold; ES, electric stimulation; EXE, exercise; CON, control.*

### Experimental Protocol

Each subject completed an experimental session which was designed to assess the effects of 30 min of low-intensity aerobic exercise on the intracortical inhibitory and excitatory circuits in M1 (Figure 1B). Transcranial magnetic stimulation (TMS) measurements were taken before exercise and at 5, 20, 40, and 60 min following 30 min of exercise or rest (denoted post 5, post 20, post 40, and post 60). In each exercise session, participants performed 30 min of low-intensity pedaling exercise at an individual load using a recumbent-type ergometer. Participants were instructed to maintain 60 rotations per min and relax their upper limbs. At the end of the exercise bout, RPE was verbally reported on a scale of 6–20. In the control session, participants rested on the ergometer instead of performing exercise. Throughout experiments, HR was monitored using a polar monitor (CS400; Polar Electro Oy, Finland), and each participant’s arousal and pleasure level were recorded using the two-dimensional mood scale (TDMS) (Sakairi et al., 2013).

### Somatosensory-Evoked Potential Measurement

Before the inhibitory-circuit experiment, somatosensory-evoked potential (SEP) was measured to determine the interstimulus interval (ISI) of SAI for each subject. Electrical stimulation (ES) was delivered using an electrical stimulator and consisted of a 0.2-ms square wave pulse. In Experiment 1, the right median nerve was stimulated using a surface-bar electrode with a cathode-proximal anode. The stimulus intensity was set at 300% of the sensory threshold (ST). The active electrode was placed at C3’, located 2 cm posterior to C3, and the reference electrode was placed at Fz. The ground electrode was placed on the skin of the left earlobe. In Experiment 2, the tibial nerve was stimulated using a surface-bar electrode on the right ankle with a cathode-proximal anode, and the stimulus intensity was set at the motor threshold (MT) of the abductor hallucis (AH). The active electrode was placed at Cz′, located 2 cm posterior to Cz, and another electrode was placed in the same position as described for Experiment 1. Five hundred stimuli were delivered at 1 Hz, and the signals were amplified and filtered. The band-pass filter was set at 3 Hz to 2 kHz. The latency of the evoked potential was measured as the time of the first positive or negative peak, referred to as N20 or P40 in Experiments 1, 2, respectively.

### Electromyographic Recording

Surface Ag/Ag Cl electrodes were placed over the muscle belly of the right first dorsal interosseous (FDI) or right tibialis anterior (TA) and soleus (SOL) in Experiments 1, 2, respectively. The raw signal was amplified and filtered with a bandpass filter of 2 Hz to 3 kHz (AB-601G; Nihon Kohden, Tokyo, Japan). Electromyography (EMG) data was collected using the Signal software (Cambridge Electronic Design, Ltd., Cambridge, United Kingdom).

### Transcranial Magnetic Stimulation Measurement

Measurement of TMS was performed using two Magstim 200 stimulators connected to a Bistim module (Magstim, Dyfed, United Kingdom), and figure-eight and double-corn coils were used in Experiments 1, 2, respectively. The coil was placed over the left M1, at the location which would elicit the largest and most consistent motor-evoked potential (MEP) in the right FDI or TA (hot spot). In Experiment 1, coil orientation was pointing posterolaterally at 45° to the sagittal plane. In Experiment 2, coil orientation was aligned so that the current in the brain flowed in a posterior to anterior direction. The location and trajectory of the coil on the scalp at the hot spot were recorded using BrainSight (Rogue Research, Canada). Resting motor threshold (RMT) was defined as the minimum intensity that elicited an MEP of at least 50 μV in five out of 10 consecutive trials in the relaxed FDI or TA muscle. The active motor threshold (AMT) was defined as the minimum intensity that elicited an MEP of at least 200 μV in 5 out of 10 consecutive trials during weak contraction of the FDI or TA muscle (5–10% of maximum voluntary contraction). Participants were given visual feedback of muscle activity and instructed to maintain tonic contraction. Stimulus intensity was set at the level required to elicit an MEP of 1 mV in Experiment 1 and 120% RMT in Experiment 2. Stimuli were applied every 5 s. To confirm whether the amplitude of single-pulse MEP had changed after exercise, 10 stimuli were delivered before each post-measurements. If the mean amplitude of MEP had changed after the exercise (more than ± 20%), we adjusted the TMS intensity to elicit same peak-to-peak amplitude as in baseline (Experiment 1: 1 mV, Experiment 2: peak-to-peak amplitude elicited by 120%RMT). In this case, both single-pulse MEP at baseline intensity and that at adjusted intensity were measured.

### Measurement of Inhibitory Circuits

We measured M1 inhibitory circuits by analyzing SICI, LICI, and SAI. The intensity of the test stimulus (TS) for all parameters was set at 1 mV and 120% of RMT in Experiments 1, 2, respectively. We assessed SICI using a paired-pulse TMS protocol (Kujirai et al., 1993). The conditioning stimulus (CS) intensity was set at 80% of AMT, and ISI was set at 2 ms. The LICI was also assessed using a paired-pulse TMS protocol (Valls-Solé et al., 1992; McDonnell et al., 2006), and CS intensity was set at the level required to elicit an MEP with a peak-to-peak amplitude of 1 mV in Experiment 1 and 120% of RMT in Experiment 2. The ISI was set at 100 ms, and SAI was assessed using combined peripheral and M1 stimulation (Tokimura et al., 2000). In Experiment 1, conditioning ES was applied to the right median nerve, and the CS intensity was set at 300% ST. In Experiment 2, electrical stimulation (ES) was applied to the right tibial nerve at the ankle, and the CS intensity was set at the MT of the AH. The ISI between electrical CS and TMS was set according to individual N20 and P40 SEP latency. Electrical stimuli preceded TMS by N20 or P40 + 2 or + 4 ms, respectively. Single-pulse MEP (TS alone), SICI, LICI and two types of SAI were stimulated randomly, and 12 stimuli were delivered for each parameter (60 stimuli). When adjusted single-pulse MEP was added to post-measurement, 72 stimuli were delivered. Calculation of SICI, LICI and SAI was carried out by expressing the conditioned MEP amplitude as a percentage of the non-conditioned single-pulse MEP amplitude for each respective time point. If adjusted single-pulse MEP was measured in post-measurement, the amplitude of adjusted single-pulse MEP was used to calculate percentage of SICI, LICI, and SAI.

### Measurement of Excitatory Circuits

To assess the M1 excitatory circuits, we measured ICF and SICF. The former was assessed using a paired-pulse TMS protocol (Kujirai et al., 1993; Ziemann et al., 1996b). The intensity of a TS in all parameters was set at 1 mV and 120% of RMT in Experiments 1, 2, respectively. The CS intensity was set at 80% AMT, and the ISI was set at 10 ms. A paired-pulse TMS protocol was also used to assess SICF (Ziemann et al., 1998b; Hanajima et al., 2002). The CS intensity was set at 90% of RMT, and was applied 1.5 or 3 ms after TS. Single-pulse MEP, ICF and two types of SICF were stimulated randomly, and 12 stimuli were delivered for each parameter (48 stimuli). When adjusted single-pulse MEP was added to post-measurement, 60 stimuli were delivered. Calculation of ICF and SICF was carried out by expressing the conditioned MEP amplitude as a percentage of the non-conditioned single-pulse MEP amplitude for each respective time point. As with inhibitory circuits, if adjusted single-pulse MEP was measured in post-measurement, the amplitude of adjusted single-pulse MEP was used to calculate percentage of ICF and SICF.

### F- and M-Wave Recording

In Experiment 3, the ratio of the F wave amplitude to the maximum M-wave amplitude for the TA and FDI was calculated to investigate the excitability of spinal anterior horn cells and motor neuron excitability before and after the low-intensity pedaling exercise. We applied ES to the right ulnar nerve at the wrist and right common peroneal nerve near the head of the fibula using a bar electrode. Stimulus intensity was increased to obtain Mmax, which was calculated by averaging five waves. To ensure maximal response, the test intensity used throughout the remaining Experiment was set at 1.2 times the intensity that evoked Mmax. Fifty F-waves were measured with the stimulus intensity set at 120% of the intensity that evoked Mmax. The ES was applied every second, with a pulse duration of 0.2 ms. The F-wave amplitude and persistence, and the ratio of F/M-wave were analyzed off-line. The intensity of ES is described in Table 1.

### Body Skin Temperature Recording

In Experiment 3, body skin temperature was also recorded to investigate the influence of changes in body temperature due to acute pedaling on central and peripheral neuromodulation. Skin temperatures were continuously measured from the left FDI, axilla, thigh and lower leg using a temperature logger (LT-8; Gram, Japan).

### Two-Dimensional Mood Scale

The TDMS was adopted to evaluate changes in psychological mood states in an efficient manner (Sakairi et al., 2013). The TDMS was developed as a psychometric scale with eight self-assessment items measured using mood-expressing words (e.g., energetic, lively, lethargic, listless, relaxed, calm, irritated, and nervous). The TDMS items consist of words corresponding to both pleasure and arousal states.

In the present study, participants were asked to complete the TDMS questionnaire before each TMS or spinal-excitability measurement. The questionnaire queried their present psychological state using a six-point Likert scale ranging from 0 = “Not at all” to 5 = “Extremely.” Participants were asked to indicate how they were feeling at the time using the scale.

### Statistical Analysis

All MEPs were expressed as peak-to-peak amplitudes. The mean MEP, RMT, HR, M-max, F-wave amplitude, F-wave persistence, F/M ratio, arousal, and pleasure level and skin temperature were analyzed using repeated-measures two-way (condition × time) analysis of variance (ANOVA) (IBM SPSS Version 18; IBM, United States). If the assumption of sphericity was violated in Mauchly’s sphericity test, the degrees of freedom were corrected using the Greenhouse–Geisser correction coefficient epsilon, and the *F* and *p*-values were then recalculated. When main effects or interactions were identified, a Bonferroni *post hoc* multiple-comparison test of significant difference was performed to identify the specific difference in factors contributing to the observed variance in the data. Specifically, each statistical value was corrected by multiplying its value by the number of comparisons. The significance level was set at *p* < 0.05. Average values are written as means ± standard error (SE).

## Results

### Exercise Intensity

Supplementary Table S1 shows the results of two-way repeated measures ANOVA of HR for all experiments. The *post hoc* test revealed that HR significantly increased after acute aerobic exercise (all experiments: *p* < 0.001), and returned to baseline level by 20 min after exercise. The HR at post 5 was significantly higher than the other time points in the exercise condition and the control condition (all experiments: *p* < 0.001) (Table 2).

**TABLE 2:** Changes in heart rate in Experiments 1–3.

			**Baseline**	**Post 5**	**Post 20**	**Post 40**	**Post 60**
Experiment1	Session A	EXE	66.7 ± 3.6	104.4 ± 3.7^**#^	68.6 ± 4.1	67.7 ± 3.5	67.6 ± 3.2
		CON	70.3 ± 3.5	69.8 ± 3.5	68.1 ± 3.6	69.2 @ 3.5	70.5 ± 3.3
Experiment1	Session B	EXE	68.3 ± 4.3	106.4 ± 3.5^**#^	70.2 ± 4.2	68.7 ± 4.1	68.1 ± 4.2
		CON	69.5 ± 3.4	70.2 ± 3.7	67.7 ± 3.3	69.4 ± 3.4	69.9 ± 3.5
Experiment2	Session A	EXE	66.8 ± 2.6	106.9 ± 2.8^**#^	68.3 ± 2.5	66.3 ± 2.4	65.8 ± 1.7
		CON	70.5 ± 2.1	68.8 ± 2.2	69.6 ± 2.6	69.3 ± 2.4	69.2 ± 2.5
Experiment2	Session B	EXE	67.6 ± 2.1	109.3 ± 2.7^**#^	68.6 ± 2.1	68.4 ± 1.9	66.9 ± 2.1
		CON	70.6 ± 1.8	70.3 ± 2.1	68.8 ± 2.3	68.5 ± 2.1	68.2 ± 2.3
Experiment3		EXE	72.1 ± 1.8	106.8 ± 2.6^**#^	75.9 ± 2.0^∗^	75.2 ± 2.0	73.1 ± 1.7
		CON	70.5 ± 2.7	72.3 ± 2.1	70.4 ± 2.3	69.3 ± 2.6	69.2 ± 2.9

*Data are presented as mean ± standard error. ^∗^*p* < 0.05 compared with other time points, ^∗∗^*p* < 0.01 compared with other time points, #*p* < 0.01 compared with control conditions. EXE, exercise (EXE); CON, control.*

In Experiment 1, the mean RPE after exercise was 11.5 ± 0.5 and 11.3 ± 1.5 in sessions A and B, respectively. In Experiment 2, the mean RPE after exercise was 11.3 ± 1.0 and 11.1 ± 1.2 in sessions A and B, respectively.

### Changes in M1 Circuits in the Non-exercised Upper Limb Area

**FIGURE 2:**
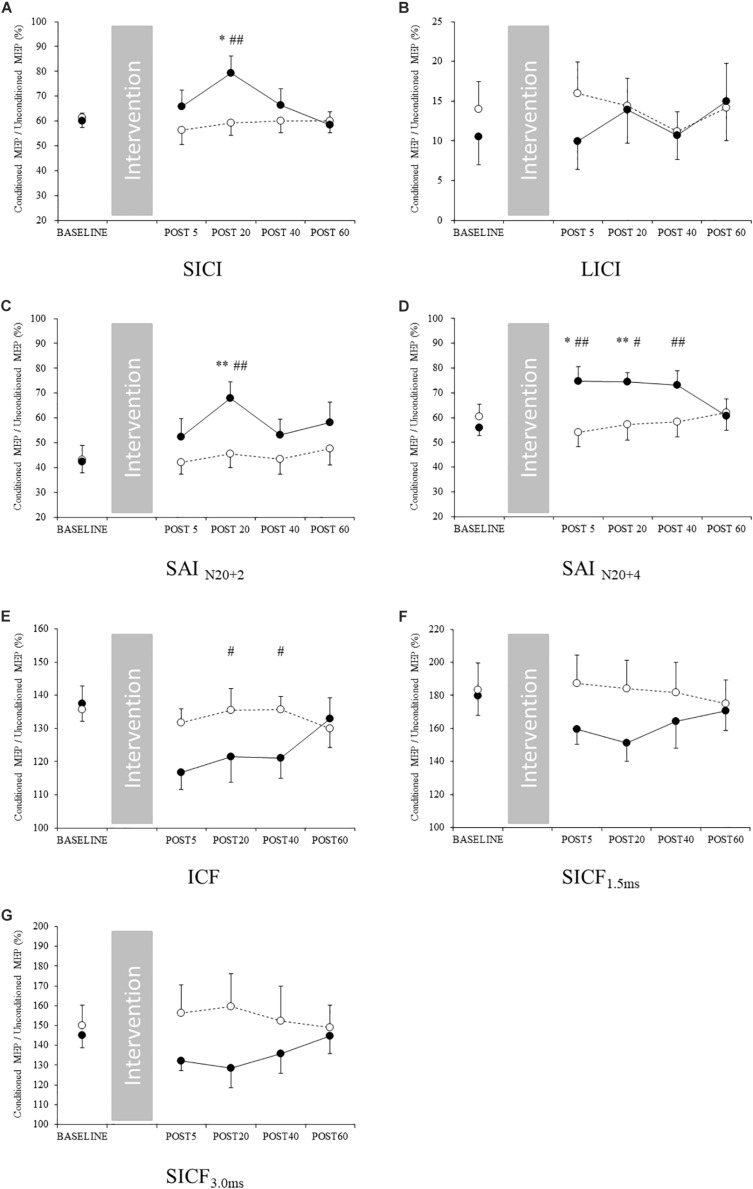
Changes in intracortical circuits of the M1 non-exercised area. Filled and open circles indicate exercise and control conditions, respectively. **(A)** short-interval intracortical inhibition, **(B)** long-interval intracortical inhibition, **(C)** short-latency afferent inhibition_*N*__20__+__2_, **(D)** short-latency afferent inhibition_*N*__20__+__4_, **(E)** intracortical facilitation, **(F)** short-interval intracortical facilitation 1.5 ms, and **(G)** short-interval intracortical facilitation 3 ms. ^∗^*p* < 0.05 compared with baseline, ^∗∗^*p* < 0.01 compared with baseline, and #*p* < 0.05 compared with control conditions, ##*p* < 0.01 compared with control conditions.

For the non-exercised upper limb, two-way repeated measures ANOVA showed significant interactions for SICI, SAI_*N*__20__+__2_, SAI_*N*__20__+__4_, and ICF (Table 3). The *post hoc* test revealed that SICI and SAI_N__20__+__2_ were significantly decreased at post 20 compared with baseline in the exercise condition (SICI: *p* = 0.038, SAI_*N*__20__+__2_: *p* < 0.001) (Figures 2A,C). In addition, at post 20, SICI in the exercise condition was significantly lower than the control condition (*p* = 0.004). At post 20, SAI was significantly lower in the exercise condition than the control condition (*p* < 0.001). The *post hoc* test revealed that SAI_N__20__+__4_ was significantly decreased at post 5 and 20 compared with baseline in the exercise condition (post 5: *p* = 0.012, post 20: *p* = 0.002) (Figure 2D). At post 5, 20 and 40, SAI_*N*__20__+__4_ was significantly lower in the exercise condition than the control condition (post 5: *p* = 0.001, post 20: *p* = 0.019, post 40: *p* = 0.009). At post 20 and 40, ICF was significantly lower in the exercise condition than the control condition (post 20: *p* = 0.019, post 40: *p* = 0.027) (Figure 2E). No main effect or interaction were observed for LICI or SICF (Figures 2B,F,G). Single-pulse MEP amplitude, TMS intensity to elicit single-pulse MEP, and RMT did not change after acute aerobic exercise (Table 3 and Supplementary Tables S2, S3).

**TABLE 3:** Results of repeated-measures analysis of variance for Experiment 1 (M1 non-exercised hand area).

		***F*-value (df)**	***p*-value**
**Experiment 1 Session A**			
Single pulse MEP	Session	0.379 (1, 14)	0.548
	Time	1.003 (4, 56)	0.414
	Session × time	1.103 (4, 56)	0.364
Single-pulse MEP (adjusted)	Session	0.237 (1, 14)	0.634
	Time	2.228 (4, 56)	0.077
	Session × time	1.144 (4, 56)	0.345
RMT	Session	2.000 (1, 14)	0.179
	Time	3.100 (2.065, 28.905)	0.059
	Session × time	0.649 (2.593, 36.307)	0.567
TMS intensity to elicit single-pulse MEP	Session	0.031 (1, 14)	0.863
	Time	0.950 (1.612, 22.562)	0.384
	Session × time	1.793 (1.768, 24.752)	0.190
SICI	Session	3.627 (1, 14)	0.07
	Time	2.286 (4, 56)	0.07
	Session × time	2.711 (4, 56)	0.039
LICI	Session	1.359 (1, 14)	0.263
	Time	1.113 (2.336, 32.708)	0.360
	Session × time	2.175 (4, 56)	0.084
SAI_*N*__20__+__2_	Session	15.588 (1, 14)	0.001
	Time	3.591 (2.428, 33.991)	0.031
	Session × time	2.602 (4, 56)	0.046
SAI_*N*__20__+__4_	Session	12.924 (1, 14)	0.003
	Time	1.352 (4, 56)	0.262
	Session × time	4.429 (4, 56)	0.004
**Experiment 1 Session B**			
Single-pulse MEP	Session	2.647 (1, 14)	0.126
	Time	1.981 (4, 56)	0.110
	Session × time	2.732 (2.181, 30.538)	0.077
Single-pulse MEP (adjusted)	Session	0.001 (1, 14)	0.980
	Time	0.469 (4, 56)	0.758
	Session × time	0.511 (4, 56)	0.728
RMT	Session	0.945 (1, 14)	0.347
	Time	2.833 (2.393, 33.507)	0.064
	Session × time	0.743 (4, 56)	0.566
TMS intensity to elicit single-pulse MEP	Session	0.039 (1, 14)	0.845
	Time	0.693 (2.526, 35.366)	0.539
	Session × time	0.845 (2.380, 33.315)	0.456
ICF	Session	5.551 (1, 14)	0.034
	Time	1.358 (2.499, 34.989)	0.272
	Session × time	2.853 (4, 56)	0.032
SICF_1__.__5_	Session	2.647 (1, 14)	0.126
	Time	1.981 (4, 56)	0.110
	Session × time	2.732 (2.181, 30.538)	0.077
SICF_3__.__0_	Session	2.883 (1, 14)	0.112
	Time	0.184 (2.143, 29.998)	0.946
	Session × time	2.181 (2.256, 31.584)	0.124

*MEP, motor-evoked potential; RMT, resting motor threshold; SICI, short-interval intracortical inhibition; LICI, long-interval intracortical inhibition; SAI, short-latency afferent inhibition; ICF, intracortical facilitation; SICF, short-interval intracortical facilitation; df, degrees of freedom.*

### Changes in M1 Circuits in the Exercised Lower Limb Area

**FIGURE 3:**
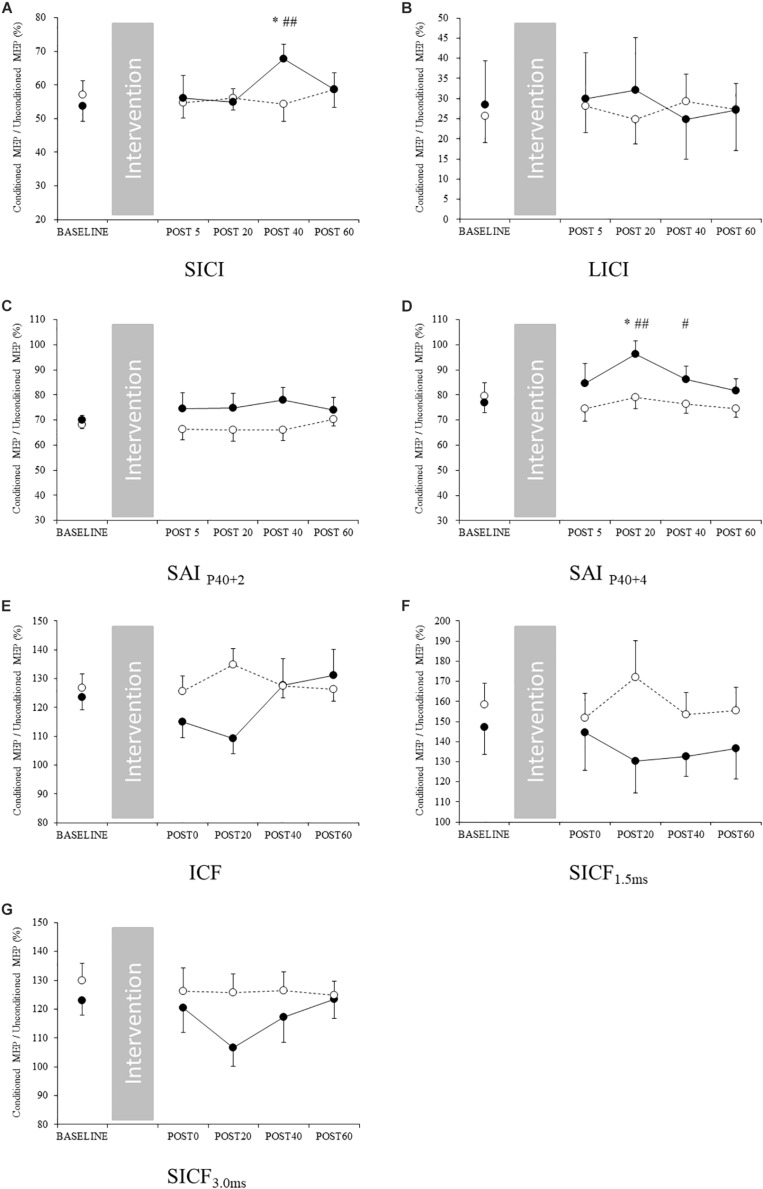
Changes in intracortical circuits in the M1 exercised area. Filled and open circles indicate exercise and control conditions, respectively. **(A)** Short-interval intracortical inhibition, **(B)** long-interval intracortical inhibition, **(C)** short-latency afferent inhibition_*P*__40__+__2_ and **(D)** short-latency afferent inhibition_*P*__40__+__4_, **(E)** intracortical facilitation, **(F)** short-interval intracortical facilitation 1.5 ms, and **(G)** short-interval intracortical facilitation 3 ms. ^∗^*p* < 0.05 compared with baseline, #*p* < 0.05 compared with control condition. ##*p* < 0.01 compared with control condition.

**FIGURE 4:**
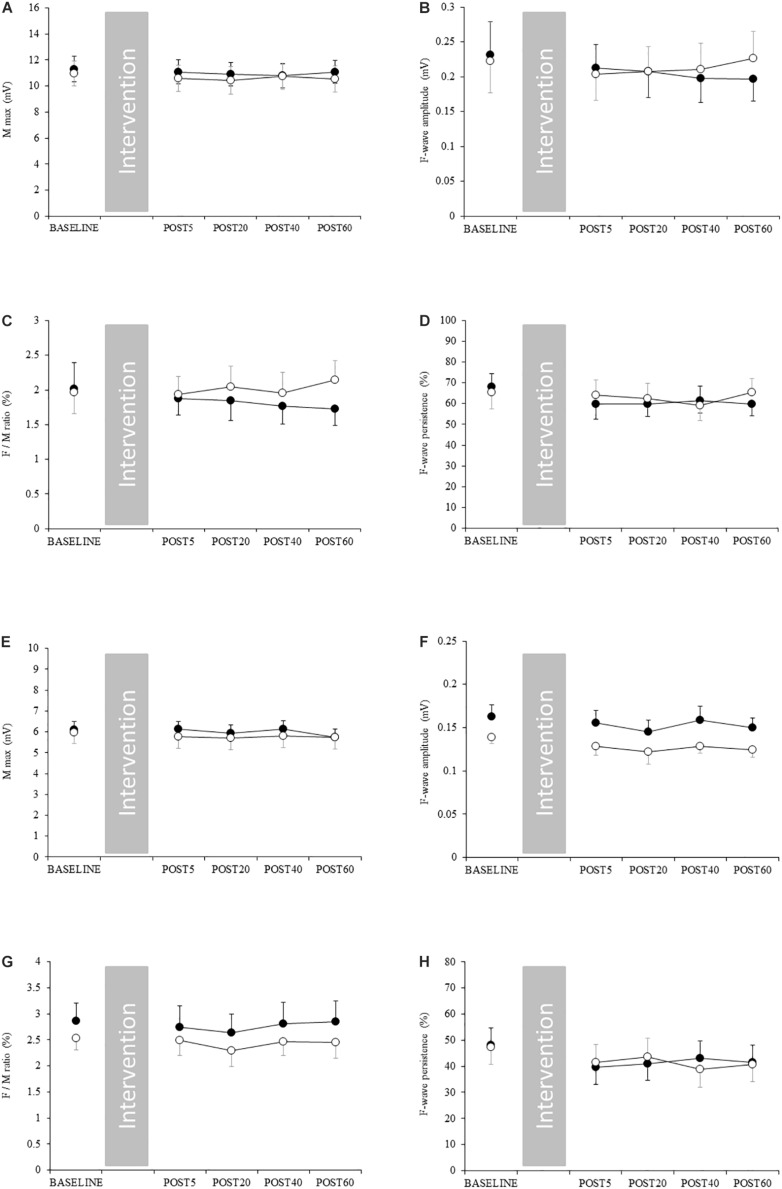
Changes in spinal excitability. Filled and open circles indicate exercise and control conditions, respectively. **(A)** Mmax, **(B)** F-wave amplitude, **(C)** F/M ratio, and **(D)** F-wave persistence in the upper limb. **(E)** Mmax, **(F)** F-wave amplitude, **(G)** F/M ratio and **(H)** F-wave persistence in the lower limb.

For the exercised lower limb, a significant interaction was identified between SICI and SAI_*P*__40__+__4_ (Table 4). The *post hoc* test revealed that SICI was significantly decreased at post 40 compared with baseline in the exercise condition (*p* = 0.031) (Figure 3A). At post 40, SICI was significantly lower in the exercise condition than the control condition (*p* = 0.018). At post 20 in the exercise condition, SAI_*P*__40__+__4_ was significantly decreased compared with baseline (*p* = 0.014) (Figure 4D). At post 20 and 40, SAI_*P*__40__+__4_ was significantly lower in the exercise condition than the control condition (post 20: *p* = 0.009, post 40: *p* = 0.014). Other parameters including single-pulse MEP amplitude, TMS intensity to elicit single-pulse MEP, and RMT did not change following acute low-intensity aerobic exercise (Figures 3B–G, Table 3 and Supplementary Tables S2, S3).

**TABLE 4:** Results of repeated-measures analysis of variance for Experiment 2 (exercised leg area).

		***F*-value (df)**	***p*-value**
**Experiment 2 Session A**			
Single-pulse MEP	Session	0.279 (1, 13)	0.606
	Time	1.098 (2.148, 27.930)	0.351
	Session × time	0.977 (2.030, 26.386)	0.391
Single-pulse MEP (adjusted)	Session	0.682 (1, 13)	0.424
	Time	0.616 (4, 52)	0.653
	Session × time	0.343 (2.277, 29.599)	0.740
RMT	Session	0.318 (1, 13)	0.583
	Time	1.206 (1.571, 20.426)	0.310
	Session × time	3.294 (2.064, 26.837)	0.071
TMS intensity to elicit single-pulse MEP	Session	0.319 (1, 13)	0.581
	Time	1.342 (4, 52)	0.266
	Session × time	2,122 (4, 52)	0.090
SICI	Session	0.007 (1, 13)	0.933
	Time	1.936 (4, 52)	0.118
	Session × time	3.319 (2.422, 31.480)	0.041
LICI	Session	0.032 (1, 13)	0.861
	Time	0.202 (1.195, 15.540)	0.703
	Session × time	1.267 (1.554, 20.206)	0.294
SAI_*P*__40__+__2_	Session	4.624 (1, 13)	0.051
	Time	0.459 (4, 52)	0.766
	Session × time	0.717 (4, 52)	0.584
SAI_*P*__40__+__4_	Session	14.741 (1, 13)	0.002
	Time	3.052 (4, 52)	0.025
	Session × time	2.911 (4, 52)	0.030
**Experiment 2 Session B**			
Single-pulse MEP	Session	0.076 (1, 13)	0.788
	Time	1.535 (2.430, 31.595)	0.229
	Session × time	0.957 (1.478, 19.215)	0.376
Single-pulse MEP (adjusted)	Session	0.028 (1, 13)	0.871
	Time	2.204 (4, 52)	0.081
	Session × time	0.346 (1.932, 25.118)	0.703
RMT	Session	0.004 (1, 13)	0.950
	Time	2.008 (2.743, 35.664)	0.135
	Session × time	1.697 (4, 52)	0.165
TMS intensity to elicit single-pulse MEP	Session	0.150 (1, 13)	0.705
	Time	0.362 (4, 52)	0.835
	Session × time	1.199 (4, 52)	0.323
ICF	Session	0.351 (1, 13)	0.564
	Time	2.476 (4, 52)	0.055
	Session × time	2.143 (4, 52)	0.089
SICF_1__.__5_	Session	0.013 (1, 13)	0.911
	Time	0.634 (4, 52)	0.640
	Session × time	0.408 (4, 52)	0.802
SICF_3__.__0_	Session	0.421 (1, 13)	0.528
	Time	1.470 (4, 52)	0.225
	Session × time	1.061 (4, 52)	0.385

*MEP, motor-evoked potential; RMT, resting motor threshold; SICI, short-interval intracortical inhibition; LICI, long-interval intracortical inhibition; SAI, short-latency afferent inhibition; ICF, intracortical facilitation; SICF, short-interval intracortical facilitation; df, degrees of freedom.*

### Changes in Spinal Excitability and Body Temperature

**FIGURE 5:**
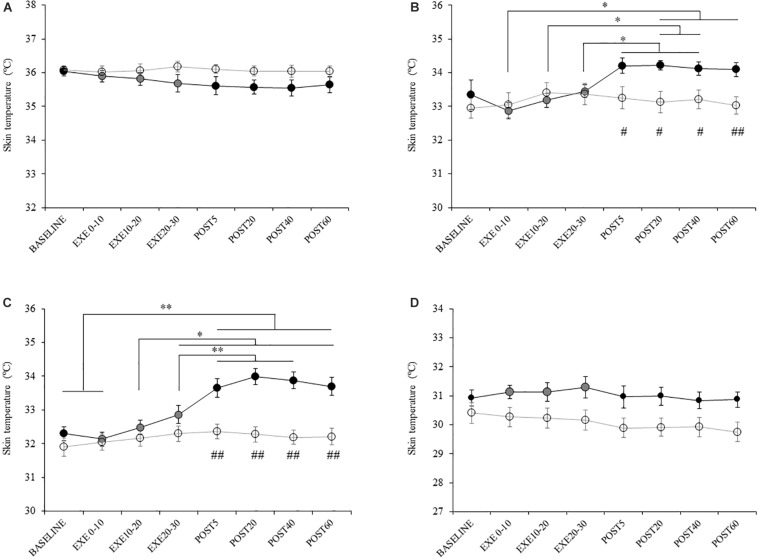
Changes in skin temperature. Filled and open circles indicate exercise and control conditions, respectively. Temperature of the **(A)** axilla, **(B)** first dorsal interosseous, **(C)** thigh, and **(D)** lower leg. ^∗^*p* < 0.05 compared with baseline, ^∗∗^*p* < 0.01 compared with baseline, #*p* < 0.05 compared with control condition. ##*p* < 0.01 compared with control condition.

Spinal excitability did not change in either the upper or lower limb, indicated by the absence of significant interaction (Figure 4 and Supplementary Table S4). Skin temperature in the FDI and thigh increased after acute aerobic exercise, as demonstrated by a significant interaction (Supplementary Table S4). The *post hoc* test revealed that skin temperature was significantly increased in the FDI at post 20 and 40 compared with during exercise (Figure 5B). Skin temperature was also significantly increased in the thigh at post 5, 20, 40, and 60 compared with that at baseline and during exercise (Figure 5C). The skin temperature in the axilla and lower leg did not change with acute aerobic exercise, indicated by the absence of a significant interaction (Figures 5A,D).

### Changes in Arousal and Pleasure Levels

Arousal levels increased following acute low-intensity aerobic exercise, indicated by a significant interaction (Supplementary Table S6). The *post hoc* test revealed that arousal level was significantly increased at post 5 in the exercise condition for all experiments (Experiment 1A: *p* = 0.029, Experiment 1B: *p* = 0.004, Experiment 2A: *p* = 0.003, Experiment 2B: *p* = 0.006, Experiment 3: *p* < 0.001) (Supplementary Table S6). In addition, the arousal level at post 5 was significantly higher than that of the control condition (Experiment 1A: *p* = 0.003, Experiment 1B: *p* < 0.001, Experiment 2A: *p* = 0.031, Experiment 2B: *p* = 0.001, Experiment 3: *p* < 0.001). Pleasure levels did not change after acute low-intensity aerobic exercise, indicated by the lack of a significant interaction (Supplementary Table S5).

## Discussion

Our study aimed to explore whether acute low-intensity aerobic exercise modulates M1 intracortical circuits in exercised and non-exercised areas. The results reveal that 30 min of low-intensity pedaling exercise suppresses SICI and SAI in M1 exercised and non-exercised areas. However, LICI and SICF do not change in response to acute aerobic exercise. In non-exercised limbs, the ICF after exercise was significantly lower than observed in the control condition but was not significantly different than baseline. Our results suggest that low-intensity aerobic exercise does not modulate corticospinal and spinal excitability, which we evaluated using single-pulse MEP, RMT and the F/M ratio.

### Changes in Corticospinal Excitability in M1 by Low-Intensity Pedaling Exercise

Single-pulse MEP and RMT in both the M1 non-exercised hand area and exercised leg area were unchanged in response to low-intensity pedaling. This suggests that acute low-intensity pedaling exercise does not modulate corticospinal excitability. In line with our results, previous studies have demonstrated that acute moderate- and low-intensity aerobic exercise does not affect single-pulse MEP or RMT (McDonnell et al., 2013; Singh et al., 2014a; Smith et al., 2014; Mooney et al., 2016; Neva et al., 2017). On the other hand, some studies have reported that high-intensity aerobic exercise increases single-pulse MEP (Ostadan et al., 2016) or decreases RMT (Coco et al., 2010), although other studies did not observe such effects (Mang et al., 2014, 2016; Stavrinos and Coxon, 2017). Taken together, the changes in corticospinal excitability that occur following aerobic exercise may depend on exercise intensity. Interestingly, Lulic et al. (2017) demonstrated that the change in input–output curve after acute moderate pedaling exercise was influenced by the physical activity level of the participant (i.e., a high physical activity level was associated with increased corticospinal excitability after exercise). Changes in corticospinal excitability may influence daily activity levels. In the present study, we did not measure physical activity levels, and enrolled participants regardless of their physical activity level. Therefore, we could not elucidate the influence of daily activity level on corticospinal and intracortical excitability.

### Changes in M1 Inhibitory Circuits Following Low-Intensity Pedaling Exercise

Thirty minutes of low-intensity pedaling exercise caused decreased SICI in both the exercised and non-exercised areas. These results are in line with previous studies (Singh et al., 2014a; Smith et al., 2014; Lulic et al., 2017; Stavrinos and Coxon, 2017). In particular, Smith et al. (2014) investigated low-intensity exercise using methods similar to our study (target HR was approximately 110 bpm), and their results strongly support our results. In addition, we found that 30 min of low-intensity pedaling exercise decreased SAI in exercised and non-exercised areas. To the best of our knowledge, this is the first study to show changes in SAI following acute aerobic exercise. It is well-known that SICI and SAI are associated with GABA_*A*_ receptor activity (Di Lazzaro et al., 2000a, 2005a,b, 2007a). Considering the results of the present and previous studies, we can conclude that aerobic exercise induces attenuation of GABA_*A*_ergic activity, regardless of exercise intensity. Interestingly, this effect is observed in non-exercised as well as exercised areas. Yamaguchi et al. (2012) demonstrated that SICI in the TA and SOL decreased following 7 min of low intensity pedaling, but was unchanged by repetitive ankle dorsiflexion or passive pedaling.

An explanation for the suppression of SICI and SAI is that aerobic exercise increases the secretion of BDNF. This neurotrophic factor plays a crucial role in promoting growth, survival and differentiation of neurons (Barde, 1994; Lindvall et al., 1994) and has been reported to suppress GABA_*A*_ergic inhibitory post-synaptic currents in the rat hippocampus (Tanaka et al., 1997; Brünig et al., 2001). Secretion of BDNF is increased by chronic (Neeper et al., 1996; Erickson et al., 2011) and acute aerobic exercise (Winter et al., 2007), and it contributes to neuroplasticity and maintenance of cognitive function (Cotman and Berchtold, 2002; Cotman and Engesser-Cesar, 2002). Additionally, Soya et al. (2007) reported that BDNF secretion in the rat hippocampus is increased by low-intensity running. Therefore, acute low-intensity aerobic exercise may have increased BDNF secretion in the cerebral cortex in this study. It is necessary to consider possible changes in GABA concentration in M1 with regards to the change in SICI and SAI after acute aerobic exercise. Mooney et al. (2016) and Stavrinos and Coxon (2017) reported that acute high- and moderate-intensity aerobic exercises do not modulate SICI at 1 ms. According to the study of Stagg et al. (2011), the magnitude of SICI at 1 ms is associated with GABA concentration in the sensorimotor area. Therefore, suppression of SICI and SAI following acute aerobic exercise may reflect changes in the activity of GABA_*A*_ receptor rather than changes in the concentration of GABA.

In addition to GABA_*A*_ergic activity, central cholinergic activity also plays a crucial role in SAI-induced inhibition (Di Lazzaro et al., 2000b, 2002, 2007b). Therefore, how aerobic exercise modulates cholinergic activity should be considered. Kurosawa et al. (1993) found that acetylcholine secretion in the rat parietal cortex increased during short-duration walking, but immediately returned to baseline upon cessation of walking. Based on this report, decreased acetylcholine secretion is unlikely to induce suppression of SAI after exercise. However, Kurosawa et al. (1993) only measured the effects of short duration exercise, whereas the exercise performed in the present study was longer and more intense. Therefore, whether acetylcholine secretion decreases following 30 min of exercise remains unclear. Taken together, decreased SAI after acute low-intensity aerobic exercise might be influenced by suppression of the SAI-related GABA_*A*_ receptor by BDNF rather than by decreased central cholinergic activity.

Another contributing factor for decreased SAI could be the excitability of S1, which also plays a crucial role in the degree of SAI. Several previous studies have reported that the modulation of S1 excitability induced by non-invasive brain stimulation leads to changes in SAI (Tsang et al., 2014, 2015; Kojima et al., 2015). In addition, Bailey et al. (2016) reported that the magnitude of SAI is correlated with the amplitude of the N20/P25 SEP component. Based on these previous studies, modulation of S1 excitability may play a crucial role in the modulation of SAI. We cannot rule out the possibility that acute aerobic exercise modulates S1 excitability. In addition, SAI is also associated with other brain regions including the thalamus (Oliviero et al., 2005) and cerebellum (CB) (Dubbioso et al., 2015). It remains unclear to what extent these areas contribute to SAI, and to what extent the activities of these areas are modulated by acute aerobic exercise. It should be noted that we used the same intensity of ES for SAI before and after exercise. Therefore, we cannot deny the possibility that responsiveness of peripheral nerves (i.e., sensory threshold) induced by ES was modulated by exercise.

We found that LICI was unchanged in response to low-intensity exercise. Suppression of LICI is well-known to be due to activity of the GABA_*B*_ receptor (McDonnell et al., 2006). Previous studies have reported inconsistent changes in LICI following acute aerobic exercise. Mooney et al. (2016) reported that acute moderate-intensity aerobic exercise caused decreased LICI; a trend that was also observed by Singh et al. (2014a) after moderate-intensity pedaling. On the other hand, Stavrinos and Coxon (2017) showed that LICI did not change in response to acute high-intensity interval training. The mechanism of acute aerobic exercise-induced change in LICI is unclear, and more detailed investigation is warranted (e.g., exercise intensity, exercise duration). However, our results indicate that acute low-intensity pedaling exercise affects GABA_*A*_ergic activity to a greater extent than GABA_*B*_ergic activity.

One peculiar finding in this study was that suppression of SICI was observed 20 and 40 min after exercise in the non-exercised and exercised areas, respectively. While previous studies have never reported this temporal difference, we speculate that the temporal modulation of the excitatory-inhibitory balance is different between upper and lower areas, or exercised and non-exercised areas. However, more detailed investigation on the temporal difference is necessary in future studies.

### Changes in M1 Excitatory Circuits Following Low-Intensity Pedaling Exercise

In the present study, we showed that ICF in the non-exercised area was lower after exercise than the control condition. However, it did not change significantly from baseline. On the other hand, ICF in the exercised area did not show any changes. After 30 min of pedaling exercise, intracortical excitatory circuits in the M1 non-exercised area were modulated but those in the exercised area were not. Previous studies have reported inconsistent results for the changes in ICF in the M1 non-exercised area following aerobic exercise. Singh et al. (2014a) reported that ICF in the M1 non-exercised upper limb area increased after moderate-intensity pedaling. By contrast, Lulic et al. (2017) demonstrated that ICF in the M1 hand area decreased after moderate-intensity pedaling. Our result was similar to the result of Lulic et al. (2017) ICF is modulated by glutamate and the *N*-methyl-D-aspartate receptor (Liepert et al., 1997; Ziemann et al., 1998a). In addition, pharmacological investigations have suggested that noradrenaline agonists increase ICF (Herwig et al., 2002; Kirschner et al., 2003), whereas selective serotonergic reuptake inhibitors reduce it Ilic et al. (2002). With regards to the changes in ICF after exercise, there is a possibility that the balance of secretion of these neuromodulators alters the effects of aerobic exercise. Although the expression of neuromodulators is elevated in line with increases in exercise intensity, the magnitude of the increase may be different. Noradrenaline release has been indicated to rapidly elevate near the lactate threshold. Therefore, in low-intensity aerobic exercise—which is below the lactate threshold—the influence of serotonin might be greater than that of noradrenaline. Furthermore, differences in the secretion of these neuromodulators might be related to mismatched results of previous studies. It should be noted that the ICF did not change from baseline in exercise condition in Experiment 1. This might be due to inter-individual variability of facilitation by ICF and to the degree of change induced by exercise.

We found that SICF did not change after exercise in either limb with any ISI, although it tended to decrease in general. Neva et al. (2017) showed that 20 min of moderate-intensity pedaling exercise enhanced SICF at 1.5 ms in the hand area of the dominant hemisphere. On the other hand, Lulic et al. (2017) reported that SICF was not modulated by 20 min of moderate-intensity aerobic exercise. Therefore, modulation of SICF by acute moderate-intensity aerobic exercise is inconsistent. Our results are similar to those of Lulic et al. (2017), although the exercise intensity in our study differed from that of previous studies, which makes direct comparisons difficult. As with ICF, SICF is also enhanced by noradrenaline agonists (Ilić et al., 2003) and suppressed by GABA_*A*_ agonists (Ziemann et al., 1996a). Therefore, acute low-intensity aerobic exercise may not cause the release of enough noradrenaline to increase M1 excitatory circuits. It has been shown that administration of selective serotonin receptor inhibitors does not affect SICF (Ilic et al., 2002), but causes decreased ICF. If low-intensity pedaling exercise upregulates serotonin secretion, it may slightly influence SICF.

### Why Does Pedaling Exercise Affect M1 Intracortical Circuits in the Non-exercised Area?

Thirty minutes of low-intensity pedaling exercise affected M1 intracortical circuits in both the exercised and non-exercised areas. It is considered that pedaling exercise affects the M1 non-exercised area via three mechanisms. Firstly, secretion of neuromodulators and neurotrophic factors generally affect the M1 hand area. For example, serotonergic neurons spread widely from the median raphe nucleus to the whole cortex. Therefore, serotonin secreted in response to pedaling exercise may affect the M1 leg area and hand area. Other neuromodulators and neurotrophic factors are also expected to affect the whole cortex. Secondly, projections from other brain areas to M1 are expected to affect intracortical circuits in the hand area. It is well-known that M1 has no anatomical connection between the hand and leg areas (Huntley and Jones, 1991). However, M1 receives inputs from many other brain regions including the premotor cortex (PM), Supplementary Motor Area (SMA), CB, basal ganglia and S1. Previous studies have reported that the representation area in the hand and leg areas may overlap in the SMA (Fink et al., 1997) and CB (Küper et al., 2012). In addition, Byblow et al. (2007) reported that the functional network between the PM and M1 in the upper limb is modulated during ankle dorsiflexion using paired-pulse TMS. There is a possibility that the influence of exercise using the leg is transferred to the hand area, and intracortical excitability in the M1 hand area may therefore be modulated by pedaling exercise. Thirdly, afferent inputs from the exercised leg limb are considered to influence the M1 non-exercised area. Feedback from group III/IV locomotor muscle afferents has been demonstrated to modulate excitability in the M1 upper-limb area (Sidhu et al., 2014). However, previous studies investigating the role of feedback from group III/IV locomotor muscle afferents on M1 excitability have only used high-intensity pedaling; thus, the influence following low-intensity pedaling exercise remains unclear. The results of Experiment 3 of the present study showed that acute low-intensity aerobic exercise increased skin temperature in both the exercised lower limb and non-exercised FDI. This is considered to occur via the whole-body circulation of the bloodstream, which increases in temperature following acute exercise. Some previous studies have demonstrated that increasing the skin temperature modulates brain activity (Egan et al., 2005), and that increasing skin temperature in the non-exercised area via acute aerobic exercise may influence intracortical circuits in the M1 hand area. However, our results should be interpreted somewhat cautiously because not all participants took part in all experiments. Additionally, we chose different stimulus intensity to elicit MEP in upper and lower limb (i.e., 1 mV in upper limb, and 120% RMT in lower limb). Thus, we could not directly compare the influence of exercise on non-exercised and exercised limbs due to the difference of stimulus intensity. To clarify the differential effects of aerobic exercise on non-exercised and exercised limbs, future studies using more controlled experimental conditions are needed.

### Changes in Spinal Excitability Following Low-Intensity Pedaling Exercise

In Experiment 3, we showed that acute low-intensity pedaling exercise did not affect spinal excitability in the exercised and non-exercised limbs. Several previous studies have reported that acute pedaling exercise does not modulate Mmax amplitude in the upper or lower limb (Motl and Dishman, 2003; Motl et al., 2003; Neva et al., 2017). Our results are consistent with these studies. On the other hand, a few studies have shown that acute pedaling decreases Mmax in the hand area (McDonnell et al., 2013; Ostadan et al., 2016). This discrepancy may be attributed to gripping the cycle ergometer handle during exercise. Because we used a recumbent-type ergometer, participants did not grip the handle in this study.

Motl and Dishman (2003) reported that acute pedaling decreases the H-reflex in the lower limb, but not in the upper limb. Their results differ slightly to our findings, which may be due to the differences in mechanism between the H-reflex and F-wave. Although both parameters are used as an index of excitability of the spinal motor-neuron pool, modulation of the H-reflex is affected by the Ia afferent. There is a possibility that acute pedaling exercise may modulate the activity of Ia afferents and the responsiveness of the spinal motor neuron-pool via sensory inputs, but not the motor neuron-pool excitability in itself.

### Clinical Implications

Previous studies have reported that acute aerobic exercise enhances neural plasticity and motor learning in healthy adults (Roig et al., 2012; Mang et al., 2014; Skriver et al., 2014; Statton et al., 2015; Snow et al., 2016; Stavrinos and Coxon, 2017), preadolescent children (Lundbye-Jensen et al., 2017; Ferrer-Uris et al., 2018) and post-stroke patients (Nepveu et al., 2017). However, these studies involved high- or moderate-intensity exercise. Those strenuous exercises are likely to reduce participant motivation or continued willingness, and involve a risk of injury, particularly in low-fitness or elderly individuals or patients. Therefore, the effectiveness of mild exercise needs further investigation. Our results show that the M1 intracortical circuits are modulated even by low-intensity pedaling exercise. A temporary decrease of intracortical GABAergic activity is known to play a key role in neural plasticity and motor learning (Perez et al., 2004; Floyer-Lea et al., 2006; Rosenkranz et al., 2007). If the decrease in SICI and SAI after exercise reflects a temporal decrease in GABAergic activity, acute low-intensity aerobic exercise may enhance neural plasticity in M1 or enhance motor learning. Future studies are required to investigate the effects of low-intensity pedaling exercise on skill acquisition or consolidation. In addition, the optimal timing for exercise to facilitate motor learning requires further investigation.

## Conclusion

Acute low-intensity pedaling exercise modulates the M1 intracortical circuits in both exercised and non-exercised areas, without causing changes to corticospinal or spinal excitability. In particular, dramatic suppression of inhibitory circuits is observed.

## Data Availability Statement

The raw data supporting the conclusions of this manuscript will be made available by the authors, without undue reservation, to any qualified researcher. Requests to access these datasets should be directed to mailto:hwd17010@nuhw.ac.jp.

## Ethics Statement

This study was conducted in accordance with the Declaration of Helsinki with the approval of the Ethics Committee of Niigata University of Health and Welfare. Informed consent was verbally obtained from all participants.

## Author Contributions

YY and DS designed the experiments. YY and SN performed the experiments. YY, DS, and KY analyzed the data. AM and HO assisted with data collection and interpretation. YY wrote the first draft of the manuscript. YY, DS, KY, HO, and AM edited the manuscript. All authors reviewed the manuscript.

## Conflict of Interest

The authors declare that the research was conducted in the absence of any commercial or financial relationships that could be construed as a potential conflict of interest.
