# Supplementary material for: Acute Low-Intensity Aerobic Exercise Modulates Intracortical Inhibitory and Excitatory Circuits in an Exercised and a Non-exercised Muscle in the Primary Motor Cortex
Source: Front Physiol. 2019 Nov 7;10:1361. doi: 10.3389/fphys.2019.01361 (PMC6853900; doi:10.3389/fphys.2019.01361)
Supplement: Supplementary file 1 [file Table_1.docx]

Table S.1 rmANOVA of HR in experiments 1, 2 and 3

|  |  | F value | p value |
| --- | --- | --- | --- |
| Experiment 1 - A | Session | 7.506 (1,14) | < 0.001 |
|  | Time | 114.990 (2.423,33.922) | < 0.001 |
|  | Session × time | 120.300 (2.013,28.175) | < 0.001 |
| Experiment 1 - B | Session | 31.395 (1,14) | < 0.001 |
|  | Time | 195.589 (2.280,31.925) | < 0.001 |
|  | Session × time | 126.274 (2.290,32.056) | < 0.001 |
| Experiment 2 - A | Session | 12.783 (1,13) | < 0.001 |
|  | Time | 168.852 (4,52) | < 0.001 |
|  | Session × time | 148.055 (4,52) | < 0.001 |
| Experiment 2 - B | Session | 15.357 (1,13) | < 0.001 |
|  | Time | 196.114 (4,52) | < 0.001 |
|  | Session × time | 95.363 (2.071,26.920) | < 0.001 |
| Experiment 3 | Session | 35.930 (1,14) | < 0.001 |
|  | Time | 86.424 (4,56) | < 0.001 |
|  | Session × time | 65.622 (4,56) | < 0.001 |

Table S.2 Changes in single pulse MEP amplitude in experiment 1 and 2.

|  |  |  |  | Baseline | | | Post 5 | | | Post 20 | | | Post 40 | | | Post 60 | | |
| --- | --- | --- | --- | --- | --- | --- | --- | --- | --- | --- | --- | --- | --- | --- | --- | --- | --- | --- |
| Experiment 1 - A | | EXE | Non-adjusted | 1.03 | ± | 0.03 | 1.09 | ± | 0.08 | 1.09 | ± | 0.07 | 0.97 | ± | 0.04 | 1.01 | ± | 0.05 |
|  |  |  | Adjusted |  |  |  | 1.09 | ± | 0.02 | 1.09 | ± | 0.03 | 0.97 | ± | 0.04 | 1.01 | ± | 0.03 |
|  |  | CON | Non-adjusted | 0.98 | ± | 0.03 | 1.00 | ± | 0.04 | 1.05 | ± | 0.05 | 1.03 | ± | 0.06 | 1.00 | ± | 0.04 |
|  |  |  | Adjusted |  |  |  | 1.00 | ± | 0.04 | 1.03 | ± | 0.03 | 1.04 | ± | 0.03 | 0.98 | ± | 0.03 |
| Experiment 1 - B | | EXE | Non-adjusted | 1.02 | ± | 0.02 | 0.99 | ± | 0.05 | 1.06 | ± | 0.04 | 1.06 | ± | 0.09 | 1.04 | ± | 0.09 |
|  |  |  | Adjusted |  |  |  | 1.02 | ± | 0.03 | 1.07 | ± | 0.03 | 1.01 | ± | 0.03 | 1.01 | ± | 0.03 |
|  |  | CON | Non-adjusted | 1.02 | ± | 0.03 | 1.04 | ± | 0.04 | 1.07 | ± | 0.06 | 1.01 | ± | 0.05 | 1.08 | ± | 0.06 |
|  |  |  | Adjusted |  |  |  | 1.04 | ± | 0.04 | 1.02 | ± | 0.04 | 1.02 | ± | 0.05 | 1.05 | ± | 0.04 |
| Experiment 2 - A | | EXE | Non-adjusted | 0.31 | ± | 0.05 | 0.30 | ± | 0.06 | 0.32 | ± | 0.05 | 0.32 | ± | 0.05 | 0.31 | ± | 0.06 |
|  |  |  | Adjusted |  |  |  | 0.31 | ± | 0.05 | 0.32 | ± | 0.05 | 0.32 | ± | 0.05 | 0.31 | ± | 0.05 |
|  |  | CON | Non-adjusted | 0.30 | ± | 0.05 | 0.29 | ± | 0.05 | 0.30 | ± | 0.05 | 0.30 | ± | 0.05 | 0.31 | ± | 0.06 |
|  |  |  | Adjusted |  |  |  | 0.29 | ± | 0.05 | 0.30 | ± | 0.05 | 0.30 | ± | 0.05 | 0.31 | ± | 0.06 |
| Experiment 2 - B | | EXE | Non-adjusted | 0.32 | ± | 0.10 | 0.31 | ± | 0.10 | 0.29 | ± | 0.06 | 0.31 | ± | 0.10 | 0.33 | ± | 0.10 |
|  |  |  | Adjusted |  |  |  | 0.32 | ± | 0.09 | 0.29 | ± | 0.07 | 0.32 | ± | 0.10 | 0.33 | ± | 0.10 |
|  |  | CON | Non-adjusted | 0.30 | ± | 0.08 | 0.34 | ± | 0.13 | 0.34 | ± | 0.13 | 0.35 | ± | 0.13 | 0.33 | ± | 0.09 |
|  |  |  | Adjusted |  |  |  | 0.31 | ± | 0.09 | 0.30 | ± | 0.09 | 0.32 | ± | 0.09 | 0.33 | ± | 0.09 |

Exercise (EXE), control (CON); mean ± standard error.

Table S.3 Changes in resting motor threshold and TMS intensity to elicit single pulse MEP.

|  |  |  | Baseline | | | POST 5 | | | POST 20 | | | POST 40 | | | | POST 60 | | |
| --- | --- | --- | --- | --- | --- | --- | --- | --- | --- | --- | --- | --- | --- | --- | --- | --- | --- | --- |
| RMT (%MSO) | |  |  | | |  | | |  | | |  | | | |  | | |
| Experiment 1 - A | | EXE | 45.7 | ± | 2.0 | 45.8 | ± | 2.0 | 46.0 | ± | 2.0 | | 45.9 | ± | 1.9 | 46.3 | ± | 1.8 |
|  |  | CON | 46.0 | ± | 2.1 | 46.1 | ± | 2.0 | 46.5 | ± | 2.0 | | 46.7 | ± | 2.0 | 46.8 | ± | 2.0 |
| Experiment 1 - B | | EXE | 47.3 | ± | 1.8 | 47.8 | ± | 1.8 | 47.9 | ± | 1.8 | | 47.9 | ± | 1.7 | 48.1 | ± | 1.7 |
|  |  | CON | 47.0 | ± | 2.0 | 47.0 | ± | 2.0 | 47.3 | ± | 1.9 | | 47.5 | ± | 1.9 | 47.7 | ± | 1.9 |
| Experiment 2 - A | | EXE | 46.2 | ± | 1.4 | 45.9 | ± | 1.5 | 45.8 | ± | 1.4 | | 45.6 | ± | 1.4 | 45.3 | ± | 1.4 |
|  |  | CON | 46.2 | ± | 1.3 | 46.1 | ± | 1.2 | 46.1 | ± | 1.3 | | 46.0 | ± | 1.3 | 46.1 | ± | 1.3 |
| Experiment 2 - B | | EXE | 45.9 | ± | 1.9 | 46.0 | ± | 1.9 | 46.1 | ± | 1.7 | | 45.9 | ± | 1.8 | 46.2 | ± | 1.9 |
|  |  | CON | 46.3 | ± | 1.8 | 46.2 | ± | 1.9 | 46.1 | ± | 1.9 | | 46.2 | ± | 1.9 | 46.2 | ± | 1.9 |
| TMS intensity to elicit single-pulse MEP (%MSO) | | | | | |  |  |  |  |  |  | |  |  |  |  |  |  |
|  | Experiment 1 - A | EXE | 57.8 | ± | 2.6 | 57.3 | ± | 2.6 | 58.0 | ± | 2.7 | | 58.1 | ± | 2.7 | 58.3 | ± | 2.7 |
|  |  | CON | 57.5 | ± | 2.7 | 57.5 | ± | 2.7 | 57.5 | ± | 2.7 | | 57.5 | ± | 2.7 | 57.6 | ± | 2.6 |
|  | Experiment 1 - B | EXE | 58.6 | ± | 2.7 | 58.9 | ± | 2.7 | 58.9 | ± | 2.6 | | 58.4 | ± | 2.7 | 58.5 | ± | 2.6 |
|  |  | CON | 58.9 | ± | 2.6 | 58.9 | ± | 2.6 | 58.9 | ± | 2.6 | | 58.9 | ± | 2.6 | 58.9 | ± | 2.6 |
|  | Experiment 2 - A | EXE | 56.1 | ± | 1.8 | 56.3 | ± | 1.9 | 55.9 | ± | 1.9 | | 56.1 | ± | 1.8 | 56.1 | ± | 1.8 |
|  |  | CON | 55.4 | ± | 1.5 | 55.4 | ± | 1.5 | 55.4 | ± | 1.7 | | 55.4 | ± | 1.7 | 55.4 | ± | 1.7 |
|  | Experiment 2 - B | EXE | 55.8 | ± | 1.6 | 56.0 | ± | 1.6 | 55.9 | ± | 1.6 | | 55.7 | ± | 1.6 | 55.7 | ± | 1.5 |
|  |  | CON | 56.3 | ± | 1.8 | 56.3 | ± | 1.8 | 56.3 | ± | 1.8 | | 56.3 | ± | 1.8 | 56.3 | ± | 1.8 |

Exercise (EXE), control (CON); mean ± standard error.

Table S4 rmANOVA of spinal excitability and skin temperature in experiment 3

|  |  | F value | p value |
| --- | --- | --- | --- |
| Spinal excitability in leg |  |  |  |
| Mmax | Session | 0.200 (1,14) | 0.662 |
|  | Time | 2.897 (2.424,33.942) | 0.060 |
|  | Session × time | 0.984 (4,56) | 0.424 |
| F-wave amplitude | Session | 12.078 (1,14) | < 0.01 |
|  | Time | 1.770 (4,56) | 0.148 |
|  | Session × time | 0.098 (4,56) | 0.983 |
| F/M ratio | Session | 1.769 (1,14) | 0.205 |
|  | Time | 0.706 (4,56) | 0.591 |
|  | Session × time | 0.066 (4,56) | 0.992 |
| F-wave persistence | Session | 0.01 (1,14) | 0.923 |
|  | Time | 2.388 (2.442,34.184) | 0.097 |
|  | Session × time | 0.785 (4,56) | 0.540 |
| Spinal excitability in hand |  |  |  |
| Mmax | Session | 0.280 (1,14) | 0.605 |
|  | Time | 1.692 (2.164,30.302) | 0.199 |
|  | Session × time | 0.849 (4,56) | 0.500 |
| F-wave amplitude | Session | 0.044 (1,14) | 0.836 |
|  | Time | 0.794 (2.126,29.766) | 0.469 |
|  | Session × time | 1.269 (1.717, 24.035) | 0.295 |
| F/M ratio | Session | 1.403 (1,14) | 0.256 |
|  | Time | 0.335 (2.444,34.218) | 0.760 |
|  | Session × time | 1.401 (1.732,24.241) | 0.264 |
| F-wave persistence | Session | 0.085 (1,14) | 0.775 |
|  | Time | 2.244 (2.457,34.404) | 0.111 |
|  | Session × time | 1.609 (4,56) | 0.185 |
| Skin temperature |  |  |  |
| Armpit | Session | 4.247 (1,14) | 0.058 |
|  | Time | 1.603 (3.056,42.790) | 0.202 |
|  | Session × time | 1.818 (3.044,42.616) | 0.158 |
| FDI | Session | 2.629 (1,14) | 0.127 |
|  | Time | 3.958 (2.159,30.225) | 0.027 |
|  | Session × time | 5.675 (2.215,31.003) | 0.006 |
| Thigh | Session | 13.574 (1,14) | 0.002 |
|  | Time | 22.791 (3.049,42.684) | < 0.001 |
|  | Session × time | 19.244 (2.357,32.998) | < 0.001 |
| Lower leg | Session | 8.405 (1,14) | 0.012 |
|  | Time | 2.788 (2.458,34.408) | 0.065 |
|  | Session × time | 1.368 (2.924,40.935) | 0.266 |

Table S.5 rmANOVA of arousal and pleasure levels in experiments 1, 2 and 3

|  |  | Arousal level | | Pleasure level | |
| --- | --- | --- | --- | --- | --- |
|  |  | F value | p value | F value | p value |
| Experiment 1 – A | Session | 9.119 (1,14) | 0.009 | 0.113 (1,14) | 0.742 |
|  | time | 5.839 (2.020,28.284) | 0.007 | 0.789 (2.138,29.936) | 0.471 |
|  | Session × time | 4.368 (4,56) | 0.004 | 0.691 (2.089,29.242) | 0.515 |
| Experiment 1 - B | Session | 7.470 (1,14) | 0.016 | 0.243 (1,14) | 0.630 |
|  | Time | 10.010 (2.415,33.810) | < 0.001 | 2.547 (2.260,31.639) | 0.088 |
|  | Session × time | 12.818 (1.987,27.812) | < 0.001 | 1.794 (2.555,35.774) | 0.173 |
| Experiment 2 - A | Session | 0.057 (1,13) | 0.815 | 0.131 (1,13) | 0.723 |
|  | Time | 11.421 (4,52) | < 0.001 | 1.013 (4,52) | 0.409 |
|  | Session × time | 4.610 (1.958,27.410) | 0.019 | 0.233 (4,52) | 0.919 |
| Experiment 2 - B | Session | 0.774 (1,13) | 0.395 | 0.207 (1,13) | 0.657 |
|  | Time | 5.843 (2.507,32.585) | 0.004 | 2.777 (2.161,28.094) | 0.076 |
|  | Session × time | 9.633 (1.986,25.813) | 0.001 | 1.293 (2.374,30.864) | 0.292 |
| Experiment 3 | Session | 2.394 (1,13) | 0.144 | 2.311 (1,13) | 0.151 |
|  | Time | 10.688 (2.353,32.949) | < 0.001 | 2.619 (2.495,34.925) | 0.076 |
|  | Session × time | 8.998 (1.668,23.354) | 0.002 | 0.652 (2.440,34.154) | 0.557 |

Table S.6 Changes in arousal and pleasure levels in experiments 1, 2 and 3

|  |  |  | Baseline | | | Post 5 | | | Post 20 | | | Post 40 | | | Post 60 | | |
| --- | --- | --- | --- | --- | --- | --- | --- | --- | --- | --- | --- | --- | --- | --- | --- | --- | --- |
| Arousal level | |  |  |  |  |  |  |  |  |  |  |  |  |  |  |  |  |
| Ex 1 | Session A | EXE | -6.9 | ± | 1.4 | -1.9 | ± | 1.1 ^* ##^ | -5.1 | ± | 0.9 ^*^ | -6.2 | ± | 1.1 | -6.1 | ± | 1.1 |
|  |  | CON | -7.5 | ± | 1.4 | -6.9 | ± | 1.1 | -7.1 | ± | 1.2 | -7.9 | ± | 1.5 | -7.4 | ± | 1.2 |
|  | Session B | EXE | -6.2 | ± | 1.1 | -1.5 | ± | 1.0 ^** ##^ | -5.3 | ± | 1.2 | -5.9 | ± | 1.3 | -5.9 | ± | 1.2 |
|  |  | CON | -6.5 | ± | 1.0 | -6.5 | ± | 1.0 | -6.8 | ± | 1.2 | -6.9 | ± | 1.2 | -6.5 | ± | 1.1 |
| Ex 2 | Session A | EXE | -7.9 | ± | 1.4 | -4.6 | ± | 1.2 ^** #^ | -8.6 | ± | 1.5 | -8.5 | ± | 1.5 | -8.5 | ± | 1.6 |
|  |  | CON | -7.8 | ± | 1.1 | -7.6 | ± | 1.1 | -7.6 | ± | 1.0 | -8.1 | ± | 1.1 | -8.1 | ± | 1.1 |
|  | Session B | EXE | -7.6 | ± | 2.1 | -1.9 | ± | 1.0 ^* #^ | -7.1 | ± | 1.2 | -7.0 | ± | 1.2 | -6.7 | ± | 1.1 |
|  |  | CON | -6.7 | ± | 1.1 | -6.8 | ± | 1.1 | -6.7 | ± | 1.1 | -6.4 | ± | 1.1 | -7.3 | ± | 1.4 |
| Ex 3 |  | EXE | -4.7 | ± | 0.9 | 0.0 | ± | 1.1 ^** ##^ | -5.3 | ± | 1.0 ^*^ | -5.8 | ± | 1.1 | -6.5 | ± | 1.2 |
|  |  | CON | -4.9 | ± | 0.8 | -6.1 | ± | 1.2 | -5.4 | ± | 1.2 | -5.4 | ± | 1.1 | -6.1 | ± | 1.2 |
| Pleasure level | |  |  |  |  |  |  |  |  |  |  |  |  |  |  |  |  |
| Ex 1 | Session A | EXE | 5.9 | ± | 1.3 | 7.1 | ± | 1.2 | 6.7 | ± | 1.2 | 7.3 | ± | 1.0 | 7.3 | ± | 0.9 |
|  |  | CON | 6.5 | ± | 1.0 | 6.5 | ± | 1.2 | 7.1 | ± | 1.0 | 6.6 | ± | 1.1 | 6.7 | ± | 1.0 |
|  | Session B | EXE | 6.5 | ± | 1.4 | 7.1 | ± | 1.4 | 6.1 | ± | 1.4 | 6.1 | ± | 1.5 | 6.1 | ± | 1.4 |
|  |  | CON | 5.7 | ± | 1.3 | 7.3 | ± | 1.1 | 6.8 | ± | 1.2 | 6.6 | ± | 1.2 | 7.1 | ± | 1.2 |
| Ex 2 | Session A | EXE | 5.7 | ± | 1.1 | 5.1 | ± | 1.0 | 6.0 | ± | 1.1 | 5.4 | ± | 1.1 | 5.7 | ± | 1.2 |
|  |  | CON | 5.3 | ± | 1.2 | 5.1 | ± | 1.3 | 5.6 | ± | 1.2 | 5.5 | ± | 1.1 | 5.2 | ± | 1.0 |
|  | Session B | EXE | 5.4 | ± | 1.2 | 7.4 | ± | 1.1 | 5.5 | ± | 1.2 | 5.9 | ± | 1.3 | 6.1 | ± | 1.2 |
|  |  | CON | 4.7 | ± | 1.3 | 6.2 | ± | 1.1 | 6.3 | ± | 1.1 | 6.1 | ± | 1.1 | 5.6 | ± | 0.9 |
| Ex 3 |  | EXE | 5.9 | ± | 1.0 | 5.9 | ± | 1.1 | 7.8 | ± | 1.1 ^*^ | 6.9 | ± | 1.2 | 7.0 | ± | 1.2 |
|  |  | CON | 6.9 | ± | 1.3 | 7.4 | ± | 1.3 | 8.1 | ± | 1.3 | 8.4 | ± | 1.3 | 8.1 | ± | 1.3 |

Exercise (EXE), control (CON); mean ± standard error. *p < 0.05 compared with baseline. **p < 0.01 compared with baseline. #p < 0.05 compared with CON condition. ##p < 0.01 compared with CON.
